# Sex-differences in fine-scale home-range use in an upper-trophic level marine predator

**DOI:** 10.1186/s40462-020-0196-y

**Published:** 2020-02-13

**Authors:** D. C. Lidgard, W. D. Bowen, S. J. Iverson

**Affiliations:** 1 Department of Biology, Dalhousie University, B3H 4J1, Halifax, Nova Scotia Canada; 2 Population Ecology Division, Bedford Institute of Oceanography, Department of Fisheries and Oceans, Dartmouth, Nova Scotia B2Y 4A2 Canada

**Keywords:** T-LoCoH, Foraging behaviour, Marine mammal, Pinniped, Movement, Foraging ecology

## Abstract

**Background:**

The distribution of prey in the ocean is spatially and temporally patchy. How predators respond to this prey patchiness may have consequences on their foraging success, and thus physical condition. The recent ability to record fine-scale movements of marine animals combined with novel home-range analyses that incorporate the dimension of time should permit a better understanding of how individuals utilise different regions of space and the consequences on their foraging success.

**Methods:**

Over a six-year study, we used T-LoCoH (Time-Local Convex Hull) home-range software to model archival GPS (Global Positioning System) data from 81 grey seals to investigate the fine-scale spatio-temporal use of space and the distribution of apparent foraging effort. Regions of home-ranges were classified according to the frequency of return visits (site fidelity) and duration of visits (intensity of use). Generalized linear mixed -effects models were used to test hypotheses on seasonal changes in foraging distribution and behaviour and the role of space-use and state on determining foraging success.

**Results:**

Male grey seals had larger home-ranges and core areas than females, and both sexes showed a contraction in home-range and core area in fall leading up to the breeding season compared with summer. Heavier individuals had smaller core areas than lighter ones, suggesting access to higher quality habitat might be limited to those individuals with greater foraging experience and competitive ability. The size of the home-range or core area was not an important predictor of the rate of mass gain. A fine-scale spatio-temporal analysis of habitat use within the home-range provided evidence of intra-annual site fidelity at presumed foraging locations, suggesting predictably in prey distribution. Neither sex nor season were useful predictors for classifying behaviour. Rather, individual identity explained much of the variation in fine-scale behaviour.

**Conclusions:**

Understanding how upper-trophic level marine predators use space provides opportunities to explore the consequences of variation in foraging tactics and their success on fitness. Having knowledge of the drivers that shape this intraspecific variation can contribute toward predicting how these predators may respond to both natural and man-made environmental forcing.

## Introduction

The distribution of prey is typically spatially and temporally aggregated, particularly in the marine environment, driven by responses to environmental changes, predation pressure and reproductive status [1, 2]. Predators respond to the spatio-temporal heterogeneity in prey distribution by allocating their time at specific locations according to the area’s intrinsic value [3]. As an example, marine predators that forage in the benthic zone, where the spatial and temporal variability in resources is often reduced, are likely to exhibit greater site fidelity than those that forage in the epipelagic zone where spatial variability is greater [4–6]. The response of predators to the distribution of prey is also governed by the costs of movement, competition and predation risks [7]. For example, in male ungulates, given the lack of paternal care, individuals trade-off forage quality with quantity when the costs of searching for high quality forage is costly due to scarcity or high interspecific competition [8]. Thus, movement patterns are the outcome of complex interactions between the environment and an individual’s state, constrained by navigational capability [8–10]. How individuals respond to variability in prey distribution can have implications for their fitness [11–14].

The concept of a home-range [15, 16] has had enduring value and has led to the development of many statistical approaches to better understand how individuals use space [17–19]. Although the home-range defines the area used to satisfy essential functions such as foraging, home-range size per se tells us little about fine scale spatio-temporal movement that ought to reflect predator-prey interactions [10, 20, 21]. Rather, it depicts a homogenous space that fails to inform on how individuals allocate time to areas within the range [22].

GPS (Global Positioning System) telemetry can provide several, high accuracy location points per hour, even for marine species that spend a large proportion of their lives submerged [23]. Incorporating high resolution spatio-temporal data in movement analyses should lead to a better understanding of fine-scale habitat use [24–27] and interactions among conspecifics [26, 28]. The inclusion of time in home-range analyses permits an examination of variation in temporal occurrence of an individual that likely reflect the heterogenous nature of resources within the home-range, and thus to define key habitats [29–31]. Areas that provide resources of relatively high quality or quantity are likely to be visited for extended periods of time (a measure of intensity of use) and re-visited (a measure of site fidelity), the frequency of which being dependent on the profitability of the resource and the rate of replenishment [4]. Fine-scale spatial and temporal analyses also provide an opportunity to identify individual specialisation in foraging tactics and implications for fitness [11, 12, 32].

The grey seal (*Halichoerus grypus*) is an upper trophic-level marine predator that inhabits temperate waters on both sides of the North Atlantic Ocean. In the Northwest Atlantic, the grey seal has a broad continental shelf distribution from the Gulf of Maine north to the Gulf of St. Lawrence with the largest breeding colony on Sable Island [33]. Grey seals on Sable Island make repeated foraging trips to shallow offshore banks on the Eastern Scotian Shelf, with a few travelling into the Gulf of St. Lawrence and south to the Gulf of Maine [34–37]. Thus, many adults exhibit behaviour analogous to a central place forager. Grey seals are size-dimorphic and previous studies have shown sex differences in their diving behaviour, diet, movement patterns and at-sea distribution [34, 35, 38–44]. Males and females, respectively, greatly reduce or fast during the breeding season relying on energy stored in the form of blubber that was acquired in the months prior to breeding [38]. Thus, both sexes can be regarded as capital breeders and as such body mass gain during the months before the breeding season necessarily reflects overall foraging success.

We used high resolution spatio-temporal GPS data and a Time-Local Convex Hull (T-LoCoH) home-range model to examine fine-scale use of space by this upper trophic-level predator and the apparent consequences of behaviour on gain in body mass (an overall measure of foraging success).

## Methods

We used a state-space behavioural switching model to estimate two behavioural states (travel vs. foraging) along each individuals GPS track. We then used T-LoCoH to estimate home-range attributes and identify fine-scale patterns of occupancy with respect to these two behaviours within the home range. By incorporating a time-scaled distance metric into the analysis, one can identify movements that have a strong temporal component and areas that are spatially connected but temporally disconnected [31, 45, 46]. If we assume that seals allocate their time at specific locations according to the area’s intrinsic value (e.g., prey patch profitability; 3), we should expect the frequency of visits to an area and the duration of stay to reflect the importance of the area to the individual. By including both behavioural and environmental attributes within a statistical framework, we aim to provide greater insight into the at-sea movements and habitat use of this large marine predator.

**Fig. 1:**
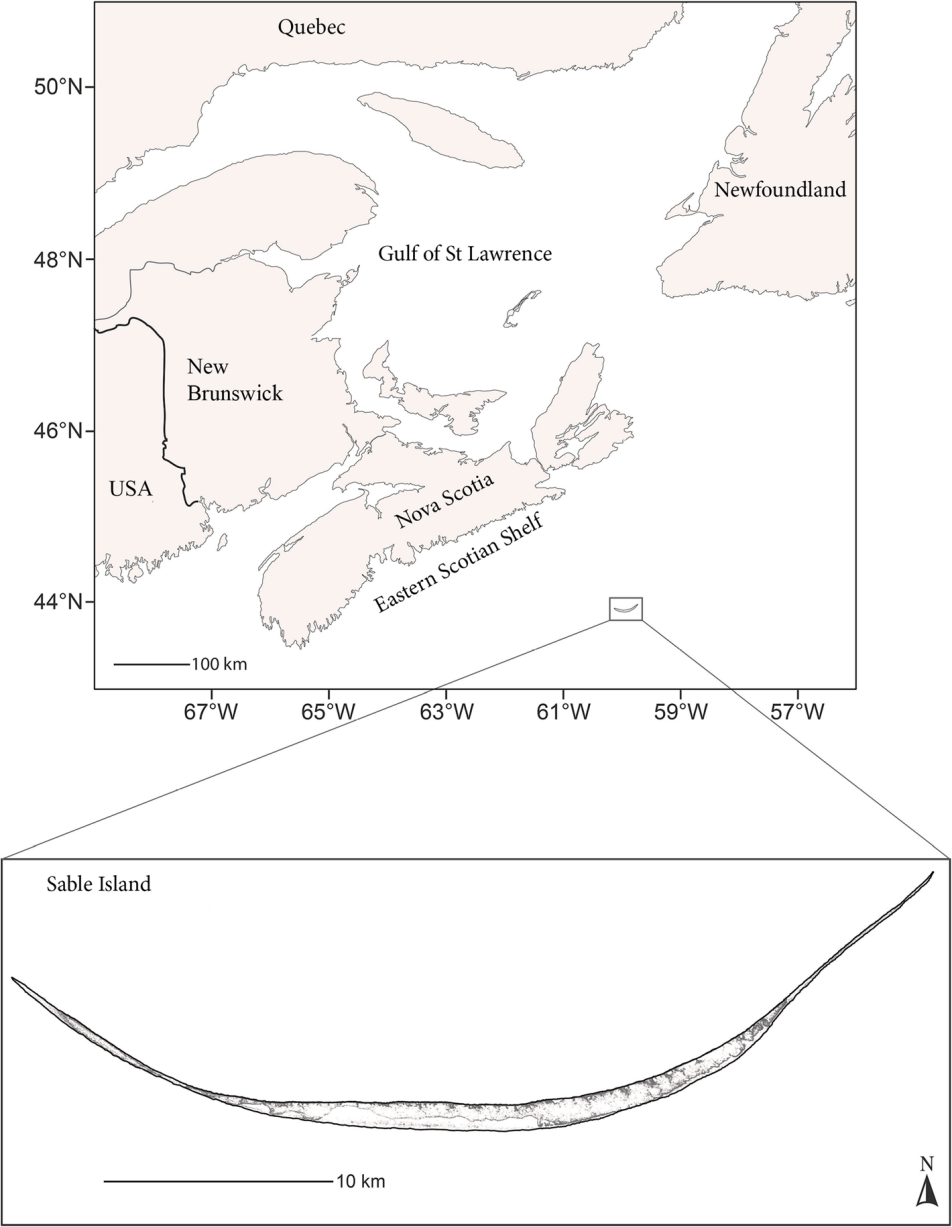
Map showing the study area and Sable Island

The study was conducted on Sable Island (Fig. 1). Sable Island is the world’s largest grey seal breeding colony and is also used as a resting site throughout the year. The Island is located near the edge of the continental shelf (43°57′00″N, 59°54′57″W) on the Scotian shelf, Northwest Atlantic Ocean. The Scotian Shelf (~ 108,000 km^2^) is comprised of a series of offshore shallow banks and basins separated by deep gullies and canyons [47] and is an important foraging area for grey seals [34]. Each year from 2009 to 2016, 15 to 20 mostly known-age adults were captured on land during the fall (September and October; 2009–2010) and in summer (June; 2011, 2013–2015) to attach electronic data loggers and telemetry tags. Tags were recovered the following December or January when the seals returned to the island to breed.

At each capture, seals were weighed using a 300 kg (± 1 kg) Salter spring balance (2009 to 2012) or a 500 kg (± 1 kg) Tractel (www.tractel.com) load cell (2013 to 2015). Individuals were immobilised using the chemical anaesthetic Telazol (intra-muscular injection; males 0.45 mg kg^− 1^, females 0.90 mg kg^− 1^) to allow attachment of tags and to accurately measure standard dorsal length. Each seal was fitted with a VHF transmitter (164–165 MHz, www.atstrack.com), a Mk10-AF Fastloc™ GPS tag (www.wildlifecomputers.com) and a Vemco Mobile Transceiver (www.vemco.com), the latter of which was part of a concurrent study as in [36]. The tag mass burden at the time of initial capture was 0.27% of body mass for males and 0.33% for females. The VHF tag was used to locate animals on Sable Island after they had returned to the breeding colony. The MK10-AF tag was programmed to record and archive a GPS location every 15 min. This corresponds to a location about every two dives on average [40]. GPS attempts were suspended when the unit was dry > 20 min and a location had been attained. The VHF transmitter was attached to the MK10-AF unit using a stainless-steel hose clamp and the whole unit was attached to the fur on the top of the head using a five-min epoxy [48].

### Body mass gain

To determine the relationships among seasonal home-range size and body mass gain, body mass gain per day relative to initial body mass was calculated for individuals whose re-capture occurred soon after arrival to the breeding colony. For adult males (*n* = 21) and non-pregnant females (*n* = 8), the date of re-capture was operationally defined as the date of their arrival given that the island was searched daily both visually and using a VHF receiver. For females that had given birth (*n* = 30), although they may have been sighted earlier, the earliest date of weighing occurred at 3 days postpartum to allow time for the female to bond with her pup and thereby minimise the risks of abandonment. Although pregnant females may arrive on the island several days prior to parturition, we did not account for mass lost by females between arrival and parturition. Most of the female’s mass loss is the result of lactation. Thus, for females weighed at 3-day postpartum, their mass on the day of parturition was estimated using average rates of female mass loss during lactation (4.1 kg day^− 1^; 49).

### Data analysis

Locations were calculated from archival GPS data using WC-DAP, a propriety software (www.wildlifecomputers.com) and archival ephemeris data (www.cddis.gsfc.nasa.gov). Locations acquired from < 5 satellites, with a residual error > 30, or considered outliers (sequential locations implying travel speed > 10 km h^− 1^) were removed (7.7% of locations) because of their lower accuracy [49, 50].

Seasonal changes in at-sea behaviour of grey seals have been observed in previous studies [34, 35, 44, 51]. Therefore, data were classified as summer (1 June to 30 September) or fall (1 October to 4 December). After ~ 4 December adults are returning to the breeding colony in increasing numbers, thus little time is spent at sea and foraging is essentially arrested. To obtain a measure of the likelihood of apparent foraging, a Hidden Markov Model (HMM) was used [52] with seal travel rate conditional upon two discrete, unobserved movement states: fast and slow movement. We assumed slow movement was associated with area restricted search (i.e., presumably foraging or resting behaviour) and characterised as high values of p (ARS), probability of area-restricted search. Low p (ARS) values suggested behaviours such as travel. Details of the HMM can be found in [36].

**Fig. 2:**
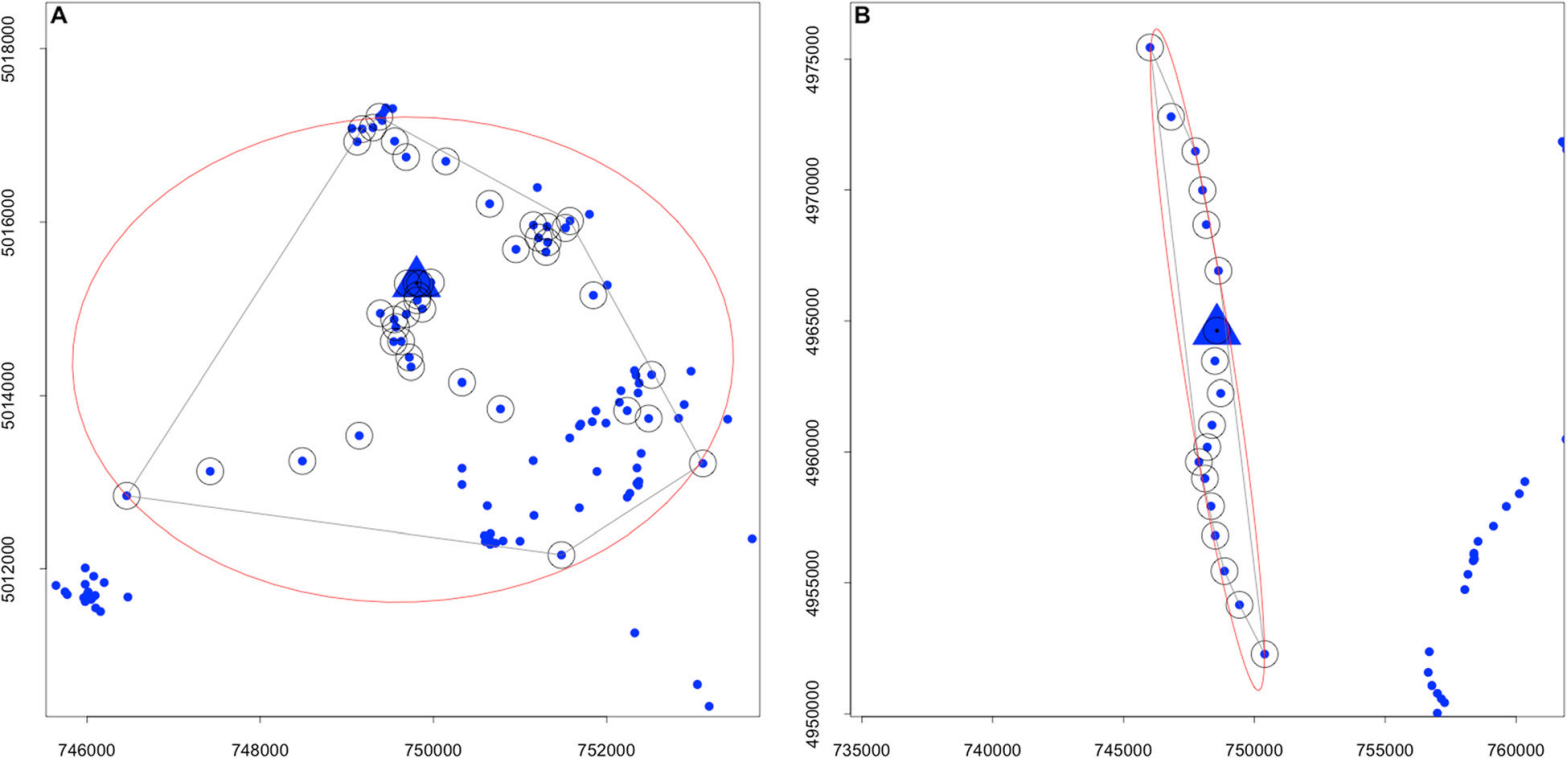
Examples of two T-LoCoH convex hulls with GPS points (•) and parent point (Δ). GPS points with an outer circle are both spatial and temporal neighbours to the parent point, while those without are spatial neighbours, but separated by time and thus excluded in the construction of the hull. The shape of the outer red ellipse is used as a proxy for behaviour: **a** – intensive, exploitative behaviour and **b** – extensive, ballistic behaviour

To quantify the use of space by individuals, we used Time-Local Convex Hull (T-LoCoH) home-range software implemented in *R* [46]. T-LoCoH is a non-parametric method for estimating home-ranges and exploring spatio-temporal patterns in movement from large GPS datasets with fine-scale temporal continuity. Utilization distributions are created from spatially and temporally defined hulls, i.e. minimum convex polygons [31, 45, 46]. Hulls are generated from each GPS location (the parent point) and neighbouring points identified according to their temporal and spatial relatedness to the parent point (Fig. 2a & b). Time is incorporated into the creation of hulls through a time-scaled distance measure (*s),* defined according to the maximum theoretical velocity of the animal, that transforms the time interval between any two locations into a third axis of Euclidean space. This measure is used to identify the number of neighbouring points within a hull that are correlated to the parent point with respect to time and space; if *s* = 0 then time plays no role in selecting neighbouring points and utilization distributions are created using the traditional space-use model. As *s* increases in value, the duration of time between successive points plays a greater role in identifying neighbouring points and creating hulls. Given that we were interested in individual variation, the chosen value of *s* was based on an evaluation of each individual’s data. We used the graphical tools available in the T-LoCoH software to visualize the relationship between the percentage of hulls that are time selected and *s*. As recommended [31], we selected a value of *s* that allowed 40 to 80% of all hulls to be time selected (the value of *s* varied between 0.03 and 0.10).

Changes in travel rate presumably reflect changes in behaviour. Thus, the number of GPS locations collected per unit area will also change. When foraging or resting, seals are likely to move within a relatively small area thus there will be more locations per unit area, whereas when travelling seals will move faster and generate fewer data points across a larger area. Therefore, hulls were generated from neighbouring data points using the *adaptive* method, as recommended by Lyons et al. [31, 46]. Using this approach, neighbour points were selected until the cumulative distance (in metres) between each neighbour and the parent point was less than or equal to a defined limit, *a*. The method is referred to as adaptive since it allows more data points to be selected, and thus more detail acquired, when the animal is moving within a small area, and fewer points when the seal is moving fast through an area in a directional manner. The choice of *a* is crucial since if the value chosen is too small, there is a heightened probability of a type I error (not including areas used by the animal) while a value too large will increase the likelihood of a type II error (including areas not used). To define *a* for each individual, we first generated a range of *a* values. To ensure we created a suitable range, we first identified the most appropriate number of nearest neighbours, *k*, as a starting point. Since we were unsure as to what was appropriate we chose a wide range (6–24 points) and then grouped them into isopleths. To identify the most suitable value of *k*, plots of isopleth area and isopleth edge:area for different values of *k* were constructed. Large jumps in the isopleth area (or edge:area) indicated a high risk of a type II error, thus values of *k* were chosen at the point when the isopleth area (and edge:area) had stabilised. For all individuals, *k* = 15 was found to be the most appropriate value. The number of nearest neighbours was then refined using the adaptive method. First, the value of *a* was calculated for *k* = 15 (a_k15_). To identify the critical value of *a*, a range of *a* values was defined centred around a_k15_. Then, for each value of *a*, nearest neighbours were selected, hulls generated and ordered from smallest to largest point density and grouped into isopleths. The same procedure for choosing the value of *k* was followed for choosing the most appropriate value of *a,* i.e. plotting isopleth area and area:edge for different values of *a*. Once values of *s* and *a* were set for each individual, several metrics associated with each hull were calculated (Table 1).

**Table 1:** Descriptions of hull metrics used to describe the behaviour of grey seals, Sable Island

Name	Description
core area	an indication of high value resource areas. Core areas were created by sorting hulls by the number of visits. Hulls were then merged until 30% of data points were contained
eccentricity	a measure of the shape of each hull, varying from 0 to 1 0 - approximates a circle, i.e. intensive, exploitative behaviour 1 - a straight line, i.e. extensive, ballistic behaviour
number of single visits to a hull	values > 1 are return visits and used as a proxy for site fidelity
normalized number of single visits to a hull	number of single visits to a hull, km^−2^
mean number of locations per visit to a hull	used as a proxy for residency
normalized number of locations per visit to a hull	mean number of locations per visit to a hull, km^−2^
probability of area-restricted search	a modelled behavioural measure that reflects the likelihood of apparent foraging

Based on [43], a seal was said to have made a return visit to a hull if the seal had been outside of the hull for more than 12 h.

Individual home-ranges based on the 95% isopleth for each season were calculated by sorting hulls according to the density of data points and then merging hulls until 95% of data points were included. To identify core areas within the home-range, we assumed areas with a higher number of repeat visits were favoured areas. We chose a 30% isopleth to identify these areas since we wanted to be conservative in selecting key resource areas and this value has been chosen in other studies (e.g., [30, 53, 54]). Thus, rather than using the number of locations to sort hulls, the visitation frequency (nsv) was used and hulls merged until 30% of data points were contained, i.e. the 30% isopleth. Given that grey seals are benthic foragers and known to spend time at offshore banks, we examined the importance of travel distance and water depth on the choice of foraging area. Although use of space could also reflect the risk of predation, adult grey seals in this population have few natural predators and therefore we hypothesize that their use of space is more likely to reflect foraging decisions. For each GPS point distance to Sable Island and bathymetry (one-minute resolution [55]) were estimated.

Linear mixed-effects models (R package lme4: [56]) were used to evaluate the importance of predictor variables (body mass, body mass gain, sex and season and their interaction) on home-range size. Seal identity was used as a random intercept. Residual plots were used to determine violations of homoscedasticity and normality. Model selection was implemented using AICc, ∆AIC_c_, weights and the evidence ratio, ER [57, 58]. Goodness of fit estimates (R^2^) for linear mixed effects models [59] are provided using R package piecewiseSEM [60]. The median absolute residual (mad) and standard error (se) are reported as the measures of variability. Statistical analyses and plots were conducted using R v3.3.2 [61].

## Results

One hundred and nineteen seals were instrumented. Of those, 102 (86%) returned to Sable Island during the following breeding season and their satellite/GPS transmitter recovered. Due to instrument malfunction, the quality of GPS data from 21 (three in 2011 and 18 in 2012) instruments were insufficient for fine-scale analyses. Thus, data were available from 81 instrumented animals (see Additional file 1). Seals were studied for an average of 192.5 ± 12.9 days. For those individuals of known age, the mean age of males (*n* = 17) and females (*n* = 53) was 17.5 ± 5.0 years (CV = 28.6%) and 24.6 ± 4.5 years (CV = 18.3%), respectively. Mean mass at deployment (mean ± sd) for males (*n* = 21) and females (*n* = 60) was 209.0 ± 36.6 kg (CV = 17.5%) and 164.0 ± 24.9 kg (CV = 15.2%), respectively. In all years, the movement of seals was confined to the Eastern Scotian Shelf and the southern Gulf of St. Lawrence with most seals using the former during repeated trips to and from Sable Island.

### Predictors of home-range

Season and sex were the best predictors of the 95% home-range size (Table 2; see Additional file 2). Body mass and a body mass-sex interaction were additional predictors in alternative models, however given the greater complexity of these models and the relatively high evidence ratio for the body mass-sex interaction model (ER = 1.79), the season-sex model was preferred. The model accounted for 58.4% of the variance (R^2^_GLMM(c)_) while the fixed effects alone accounted for 30.6% (R^2^_GLMM(m)_). The size of the 95% home-range area was greater in male grey seals than females, and for both sexes, the home-range contracted during the fall period prior to the breeding season (Table 3).

**Table 2:** Linear mixed-effects models for 95% home-range area of grey seals, Sable Island, Nova Scotia, 2009–2011 and 2013–2015

Model	K	AIC_c_	∆AIC_c_	w	LL
Bm + Sn + Sx + Bm * Sx	7	−34.1	0	0.36	24.5
Bm + Sn + Sx	6	−32.9	1.16	0.20	22.8
Sn + Sx	5	−32.8	1.24	0.19	21.7
Bm + Sn + Sx + Sn * Sx + Bm * Sx	8	−31.8	2.27	0.12	24.5
Bm + Sn + Sx + Sn * Sx	7	− 30.8	3.27	0.07	22.9
Sn + Sx + Sn * Sx	6	− 30.7	3.35	0.07	21.7
Sn	4	−15.3	18.8	0	11.8
Bm + Sn	5	−14.9	19.2	0	12.7
Bm + Sx + Bm * Sx	6	−3.40	30.7	0	8.03
Bm + Sx	5	−2.90	31.1	0	6.72
Sx	4	0.20	34.3	0	4.04
~	3	13.8	47.9	0	−3.82
Bm	4	15.7	49.8	0	−3.71

**Table 3:** The 95% home-range (km^2^) for male and female grey seals during the summer and fall periods, Sable Island, Nova Scotia, 2009–2011 and 2013–2015

	Summer			Fall		
sex	median, km^2^	mad	n	median, km^2^	mad	n
Male	3260	788	10	2204	1149	20
Female	2092	1283	37	1171	511	59

*mad* median absolute deviation.

Fixed effects of each candidate model are body mass at deployment (Bm), season (Sn) and sex (Sx). Variables for model selection are Akaike’s information criteria (AICc, ∆AIC_c_), Akaike weights (w) and log likelihood (LL). For all models, N_seals_ = 81, N_obs_ = 126.

Body mass, season, sex and the interactions body mass * sex and season * sex were the best predictors of the size of the core area (Table 4; see Additional file 3; R^2^_GLMM(c)_ = 18.5%; R^2^_GLMM(m)_ = 18.5%). Males had a larger core area than females and in both sexes the core area contracted during the fall period with males experiencing a higher magnitude of change than females (Table 5). The core area decreased in size with body mass in both sexes.

**Table 4:** Linear mixed-effects models for size of the core area of grey seals, Sable Island, Nova Scotia, 2009–2011 and 2013–2015

Model	K	AIC_c_	∆AIC_c_	w	LL
Bm + Sn + Sx + Sn * Sx + Bm * Sx	8	92.7	0	0.64	37.7
Bm + Sn + Sx + Bm * Sx	7	96.0	3.38	0.12	−40.5
Bm + Sn + Sx + Sn * Sx	7	96.2	3.51	0.11	− 40.6
Bm + Sn + Sx	6	97.7	5.03	0.05	− 42.5
Sn + Sx + Sn * Sx	6	98.3	5.66	0.04	−42.8
Sn + Sx	5	100.2	7.54	0.02	−44.8
Bm + Sx + Bm * Sx	6	100.8	8.16	0.01	−44.1
Sn	4	101.7	9.02	0.01	−46.7
Bm + Sx	5	102.0	9.38	0.01	−45.8
Bm + Sn	5	103.5	10.81	< 0.01	−46.5
Sx	4	106.3	13.65	< 0.01	− 49.0
~	3	107.3	14.61	0	− 50.5
Bm	4	108.3	15.66	0	−50.0

**Table 5:** The 30% core home-range (km^2^) for male and female grey seals during the summer and fall periods, Sable Island, Nova Scotia, 2009–2011 and 2013–2015

	Summer			Fall		
sex	median, km^2^	mad	n	median, km^2^	mad	n
Male	379.0	213.0	10	168.3	81.3	20
Female	230.0	235.6	37	132.6	62.5	59

The core area is defined as the 30% isopleth based on number of visits to a hull. Fixed effects of each candidate model are body mass at deployment (Bm), season (Sn) and sex (Sx). Variables for model selection are Akaike’s information criteria (AICc, ∆AIC_c_), Akaike weights (w) and log likelihood (LL). For all models, N_seals_ = 81, N_obs_ = 126.

The core area is defined as the 30% isopleth based on number of visits to a hull. Mad = median absolute deviation.

### Predictors of body mass gain

During the combined summer and fall periods, males (n = 21) gained 0.30% of their initial mass per day (mad = 0.13) while females (*n* = 38) gained 0.22% per day (mad = 0.11). Sex was the best predictor of body mass gain (R^2^ = 14%; Table 6 a & b; see Additional file 4). Neither, the 95% home-range (Table 6 a) nor core-area (Table 6 b) were good predictors of mass gain.

**Table 6:** Linear models for relative body mass gain (a: 95% home range and sex; b: core area and sex) of grey seals, Sable Island, Nova Scotia, 2009–2011 and 2013–2015

Model	K	AIC_c_	∆AIC_c_	w	LL
a					
Sx	3	− 197.1	0	0.67	101.8
Hr + Sx	4	− 195.0	2.11	0.24	101.9
Hr + Sx + Hr * Sx	5	−192.6	4.48	0.07	101.9
~	2	− 189.6	7.54	0.02	96.9
Hr	3	− 187.4	9.77	0.01	97.0
b					
Sx	3	−197.1	0	0.58	101.8
Ca + Sx + Ca * Sx	5	−195.2	1.91	0.22	103.2
Ca + Sx	4	− 194.8	2.29	0.18	102.0
~	2	−189.6	7.54	0.01	96.9
Ca	3	−187.6	9.48	0.01	97.0

Fixed effects of each candidate model are a) 95% home-range area (Hr) and sex (Sx); b) core area (Ca) defined as the 30% isopleth based on number of visits to a hull, and sex (Sx). N_seal_ = 59. Variables for model selection are Akaike’s information criteria (AICc, ΔAIC_c_), Akaike weights (w) and log likelihood (LL)

### Spatio-temporal use within the home-range

**Fig. 3:**
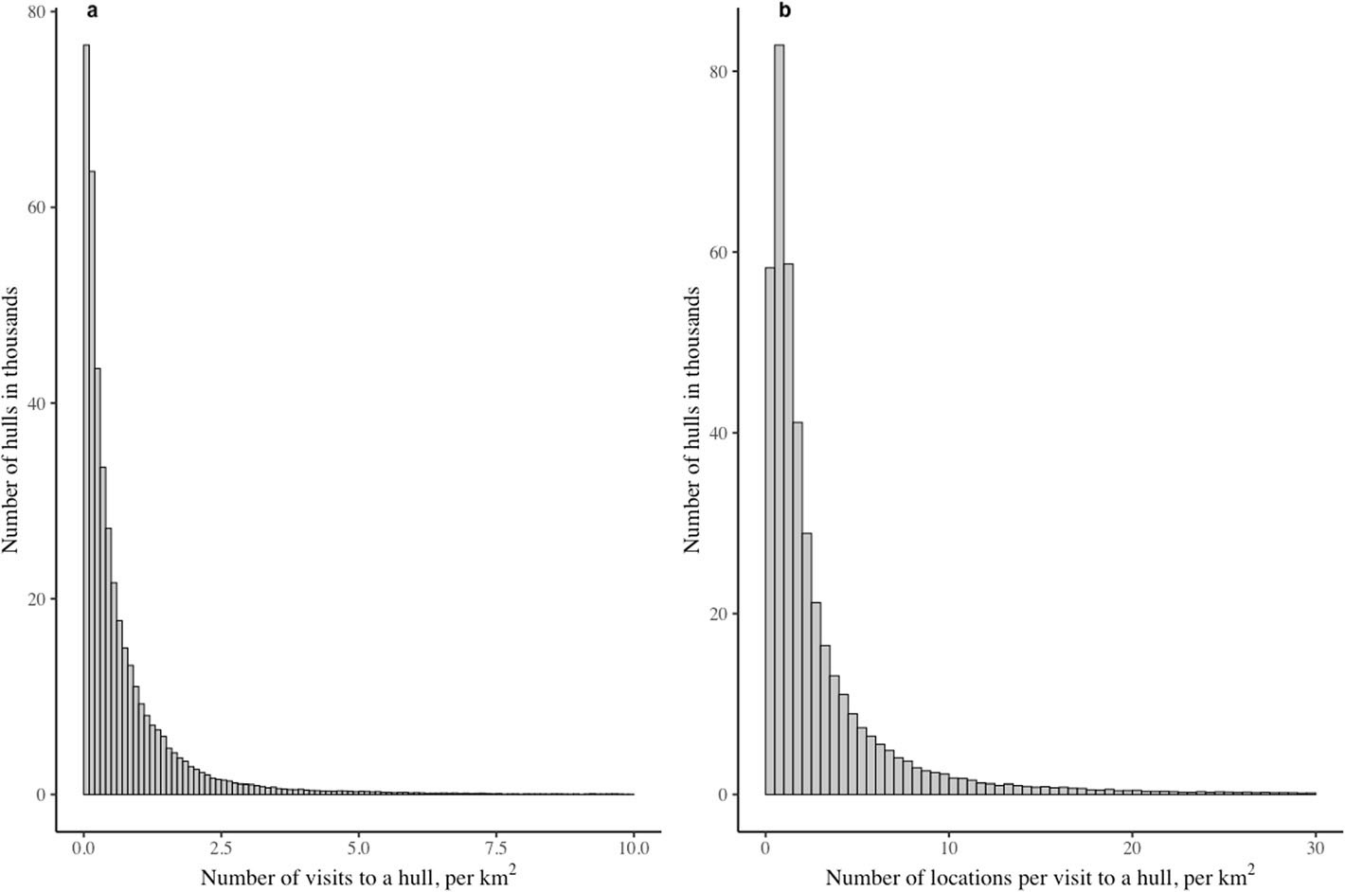
A histogram of (**a**) the normalised number of visits to a hull per km^2^ and (**b**) the normalised number of locations within a hull per km^2^ for grey seals (*n* = 81), Sable Island, Nova Scotia

Grey seals often returned to areas previously visited within their home range. The frequency distribution of visits to locations was strongly positively skewed suggesting preference for some areas over others (Fig. 3a). The amount of time spent at a location (Fig. 3b) was also strongly positively skewed, indicating differential use and suggesting that some areas were preferred over others. Overall, the broad distributions of both frequency and duration of visits to hulls suggest that seals were exhibiting multiple behaviours while at sea.

To examine this hypothesis, we defined six behavioural categories based on visit frequency and duration to a hull (see Additional file 5). We used the first and third quartiles of the normalized number of visits to a single hull (see Table 1 for definition; 0.14 and 0.89 visits per km^2^, respectively) to split behaviour into three categories: infrequent, frequent and very frequent visits to the same hull. Then, each of the three visitation frequency categories was split again according to the median of the normalized number of locations per visit (see Table 1 for definition; 0.85, 1.46, and 3.31 locations per km^2^, respectively). Hulls within the vicinity of Sable Island had high frequency of return visits of short duration presumably representing short repeated movements of seals near the island. To avoid biasing data with these frequent short trips, GPS points that were within 15 km of Sable Island were excluded from this analysis.

**Fig. 4:**
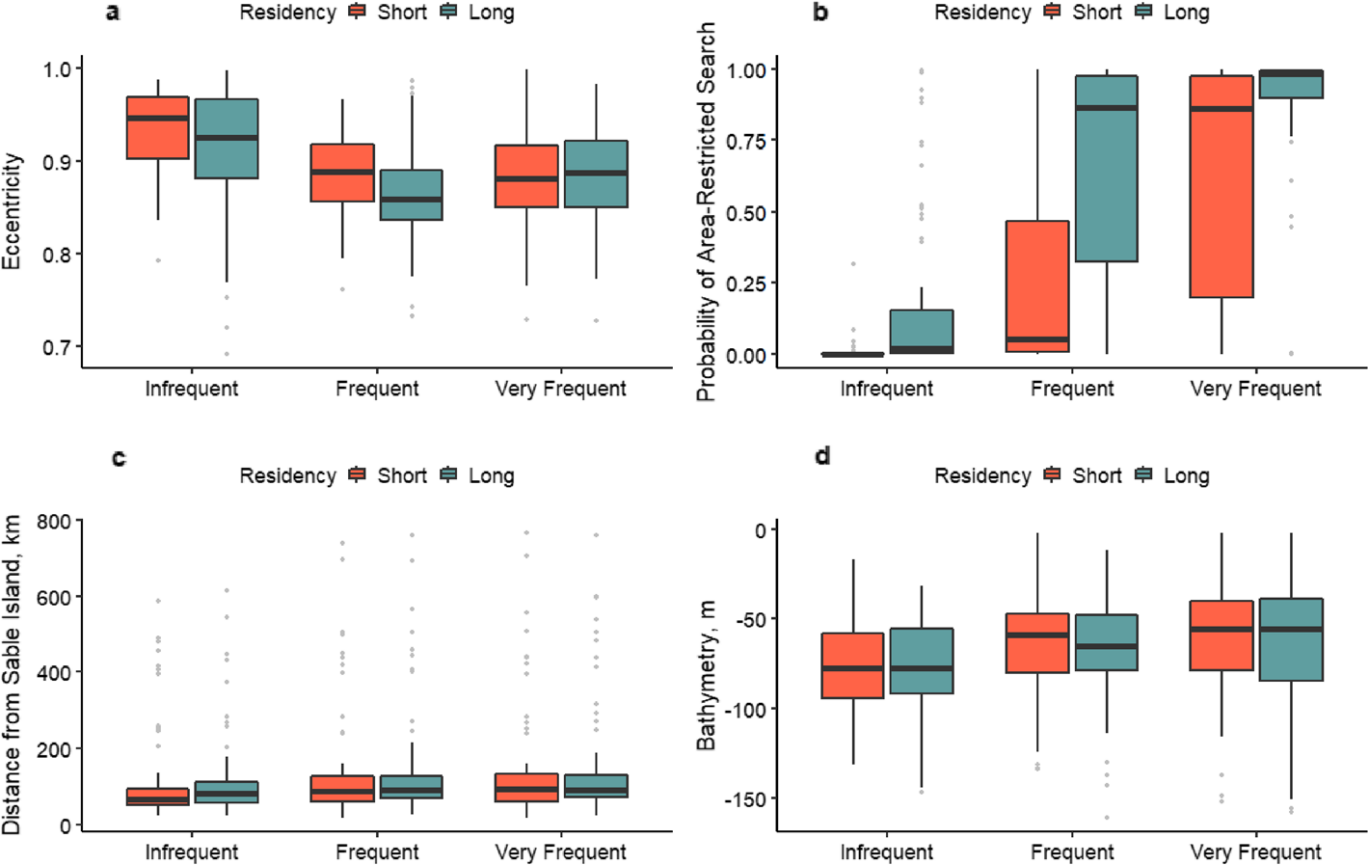
Metrics describing the characteristics of hulls for grey seals (*n* = 81), Sable Island, Nova Scotia. **a** , eccentricity; **b** , probability of area-restricted search; **c** , distance from Sable Island; **d** , bathymetry. Hulls were classified according to the number of visits to and number of locations within a hull

Seal behaviour in a hull, represented by eccentricity (Fig. 4a) and modelled behavioural state (Fig. 4b), showed wide variation both within and among the six behaviour categories. The shape of hulls for which seals visited infrequently (i.e., those in behaviour categories 1 and 2), tended to be more eccentric suggesting travel. The probability of area-restricted search was highest for those hulls within category 6 which had the highest frequency of return visits and the longest duration within the hull (see Additional file 5). Hulls in categories 4 and 5 also had relatively high values of apparent foraging, but there was much greater variability suggesting other behaviours as well. We hypothesized that hulls with the fewest return visits (behavioural categories 1 to 2) were most likely associated with travel while those with the most return visits (categories 5 to 6) were most likely associated with foraging. For both sexes, hulls within categories 5 to 6 tended to occur over offshore banks relatively close to Sable Island (Additional file 6 a & b).

Much of the seals’ activities occurred within ~ 150 km of Sable Island (Fig. 4c). The depth of water for hulls visited by seals fell between 50 m and 75 m, indicating that seals spent considerable time at shallow offshore banks (Fig. 4d). Variability in the distance from Sable Island and bathymetry was high within, but less variable among categories (Fig. 4c and d).

**Fig. 5:**
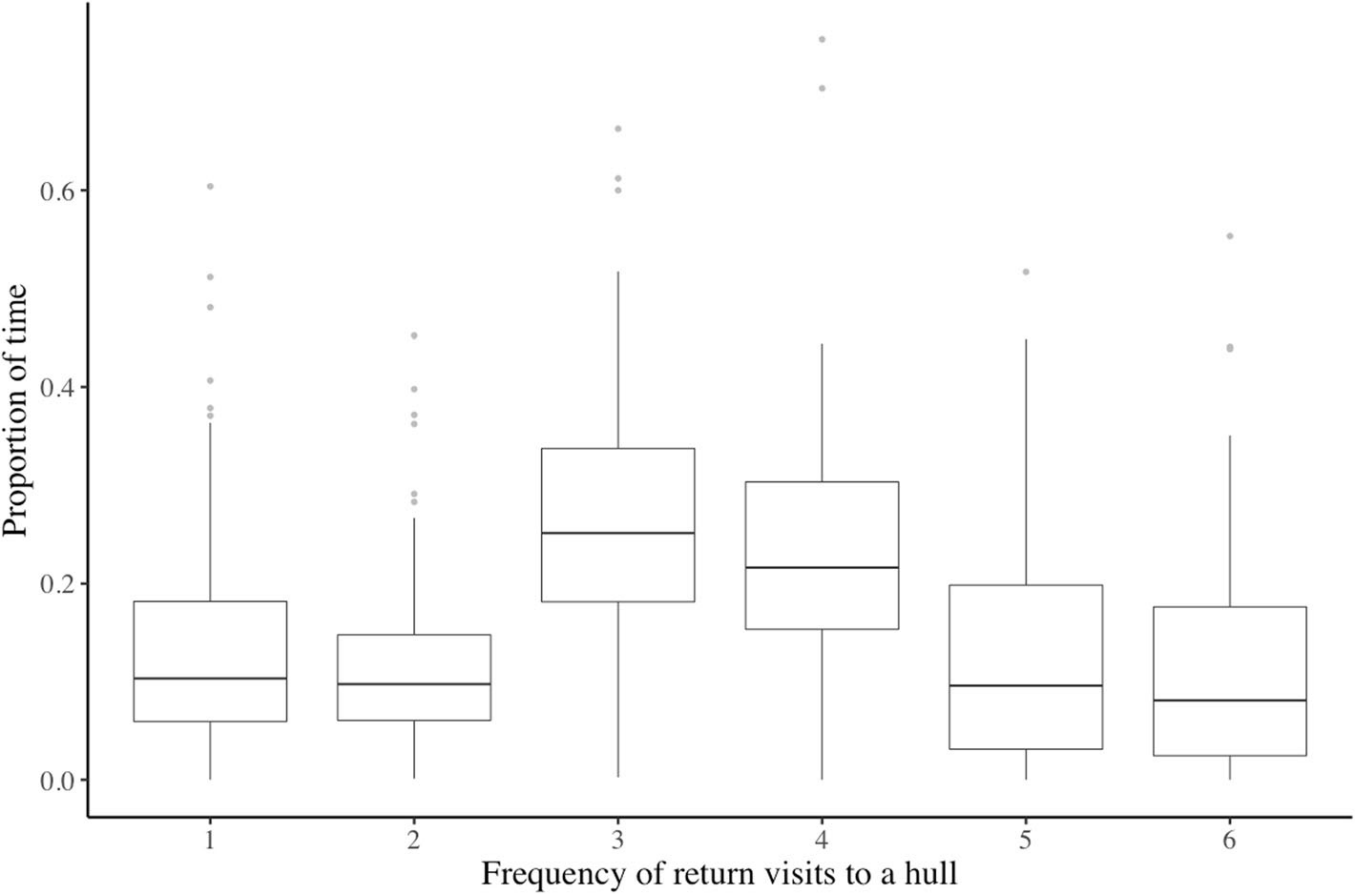
Proportion of time grey seals (*n* = 81) spent in hulls within behavioural categories 1 to 6, 2009–2011 and 2013–2015

The proportion of time seals spent in hulls within each of the six categories appeared to be governed by the frequency of return visits to the hull (Fig. 5; F_2,237_ = 188.3, *p* < 0.001) and was not driven by sex (F_1,237_ = 0.300, *p* = 0.585). Seals spent the greatest proportion of time in hulls with frequent re-visitation (i.e., categories 3 and 4; median = 0.627, mad = 0.132; Tukey post-hoc, all *p* values < 0.0001), and the least proportion of time in hulls with infrequent re-visitation (i.e., categories 1 and 2; median = 0.103, mad = 0.078; Tukey post-hoc, all p values < 0.0001). There was also a significant seasonal interaction (F_2,258_ = 10.5, p < 0.001). First, the pattern observed above was apparent in both the summer and fall seasons (Tukey post-hoc, all p values < 0.0001). However, during the summer seals spent a greater proportion of time in hulls with infrequent re-visitation compared with the fall (summer: median = 0.160, mad = 0.120; fall: median = 0.094, mad = 0.065), and a lower proportion of time in hulls with very frequent re-visitation (summer: median = 0.135, mad = 0.178; fall: median = 0.178, mad = 0.212; Tukey post-hoc, all p values < 0.05).

The duration of time spent in hulls of categories 5 and 6 (i.e., very frequent visitation) was highly variable among individuals (median = 5.5 h; mad = 3.5 h). Body mass, season and sex were included in models that were equally as useful at predicting the time spent in categories 5 and 6 as the intercept model, thus we chose the most parsimonious model (Table 7: see Additional file 6 a). Time spent in hulls associated with categories 5 to 6 was also not a good predictor of the relative rate of mass gain. In agreement with earlier analyses, sex was the best predictor (Table 8: see Additional file 6 b).

**Table 7:** Linear models for proportion of time spent in hulls within categories 5 to 6 (very frequent visitation) for grey seals, Sable Island, Nova Scotia, 2009–2011 and 2013–2015

Model	K	AIC_c_	∆AIC_c_	w	LL
Bm	3	−90.1	0	0.21	48.2
Bm + Sn	4	−89.8	0.31	0.18	49.1
~	2	−89.5	0.64	0.15	46.8
Sx	3	−88.8	1.38	0.1	47.5
Sn	3	−88.5	1.64	0.09	47.4
Bm + Sx	4	−88.1	2.07	0.07	48.2
Sx + Sn	4	−87.9	2.25	0.07	48.1
Sn + Sx + Bm	5	−87.7	2.44	0.06	49.1
Sn + Sx + Sn * Sx	5	−87.0	3.14	0.04	48.8
Bm + Sn + Sx + Sn * Sx	6	− 86.6	3.54	0.04	49.7

Fixed effects of each candidate model are body mass at deployment (Bm) and sex (Sx), N_seals_= 81. Variables for model selection are Akaike’s information criteria (AICc, ΔAIC_c_), Akaike weights (w) and log likelihood (LL)

**Table 8:** Linear models for relative body mass gain and proportion of time spent in hulls within categories 5 to 6 for grey seals, Sable Island, Nova Scotia, 2009–2011 and 2013–2015

Model	K	AIC_c_	∆AIC_c_	w	LL
Sx	3	− 202.9	0	0.68	104.7
Sx + t_5,6_	4	−200.7	2.21	0.23	104.7
Sx + t_5,6_ + Sx * t_5,6_	5	− 198.6	4.37	0.08	104.8
~	2	−194.7	8.23	0.01	99.5
t_5,6_	3	− 193.1	9.79	0.01	99.8

Fixed effects of candidate model are time spent in hulls within categories 5 to 6 (t_5,6_), N_seals_ = 59. Variables for model selection are Akaike’s information criteria (AIC_c_, ∆AIC_c_), Akaike weights (w) and log likelihood (LL)

**Fig 6:**
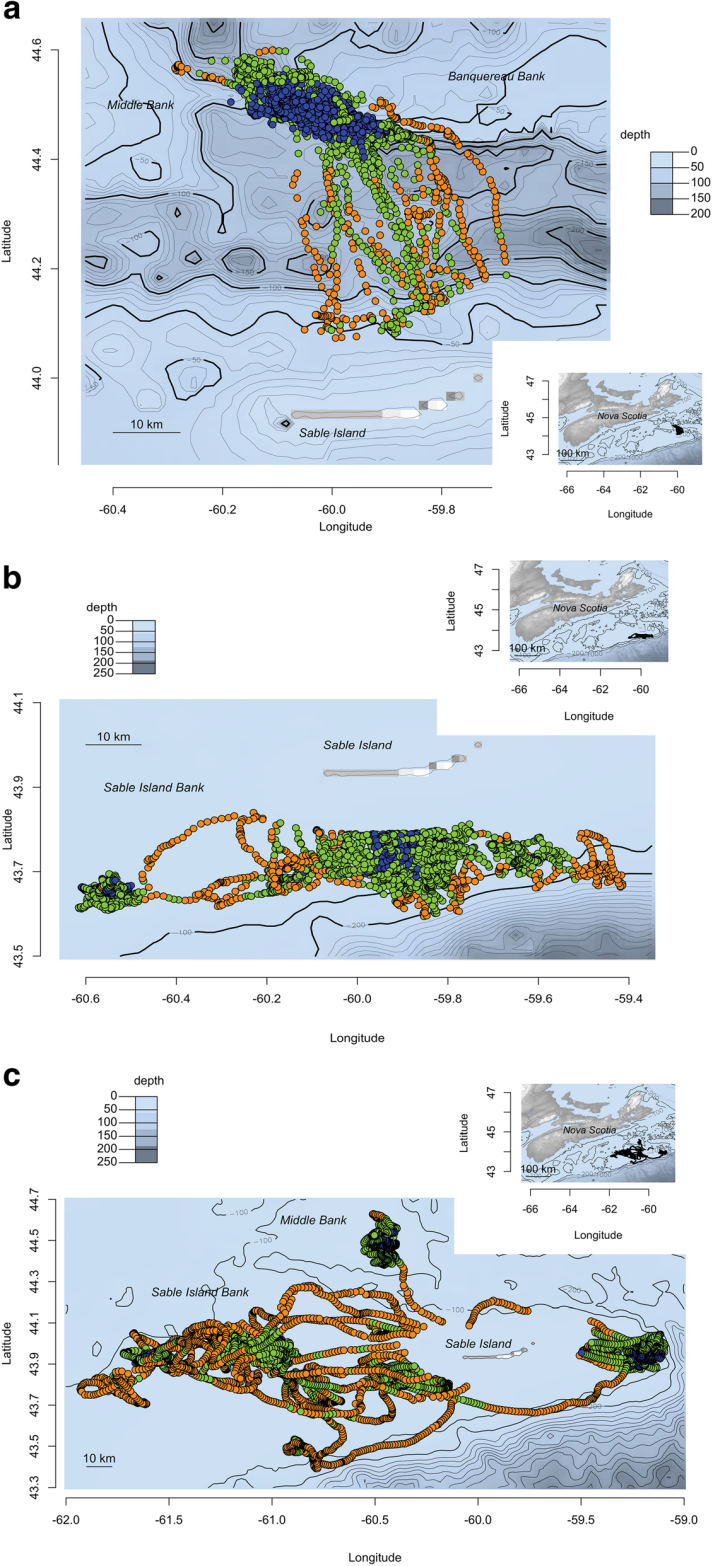
**a-c** Examples of movement tracks for three female grey seals on the Eastern Scotian Shelf. Each map shows both broad (map insert) and detailed geographical views of the movement of the seal. Due to their density, GPS locations within 15 km of Sable Island are not shown on the map. Bathymetry contours (depth, m) are given. GPS locations are coloured according to the behavioural categories 1 to 2 (infrequent visits to a hull; dark orange), 3 to 4 (frequent visits to a hull: green) and 5 to 6 (very frequent visits to a hull; blue) (see text for additional description of behavioural categories)

We plotted contrasting movement tracks of three adult female grey seals to illustrate the geographical distribution of hulls categorized by behaviour. Hulls within behavioural categories 1 and 2 (infrequent visitation), occurred closer to Sable Island and tended to form linear sequences of locations (Fig. 6 a-c). In contrast, hulls within behaviour categories 5 and 6 (very frequent visitation), tended to cluster over offshore banks (e.g., Middle, Banquereau and Sable Island Banks) (Fig. 6 a-c). Hulls within categories 3 and 4 (frequent visitation) likely comprised multiple behaviours with some hulls occurring in linear sequences while others appear clustered.

## Discussion

We collected GPS location data every ~ 15 min, which represented a location every two to three dives. Such high-resolution data provided the opportunity to examine movement and habitat use of a marine predator on a fine-scale. To take advantage of these high-resolution data, we used an adaptive non-parametric home-range model, T-LoCoH [31], that incorporates time into the construction of minimum convex hulls around each GPS point. The inclusion of time allowed us to identify areas within the home-range according to their intensity and pattern of use, both of which may serve as an indication of variation in habitat quality [30, 62].

The 95% home-range size of male and female grey seals was considerably smaller in this study compared with a previous study on the same population [63]. However, the earlier study used location data derived from ARGOS to estimate movement trajectories and fixed-kernel home-range estimators, both of which are known to overestimate home-range size [46, 49, 64]. Nevertheless, it is also possible that the increase in the size of the Sable Island grey seal population [33] and associated increase in intraspecific competition [51, 65] may have contributed to changes in home-range size over time.

The ability to assess the extent and variability of space use in pinnipeds is governed by the duration of deployments and the number of animals in the study. Pinnipeds undergo an annual moult thus limiting the length of deployments and the ability to measure the full extent of individual movements. In this study, the majority of individuals wore tracking instruments for the period immediately following the moult and up to the start of the breeding season, providing the maximum recording period (6–7 months). Recent studies have highlighted the need for large sample sizes (10 to 100) when estimating utilization distributions due to the high variability in movement behaviour among individuals of a given species [66–68]. In this study, instruments were deployed on 15 to 20 individuals each year for a seven-year period to provide an adequate sample size to account for individual variation, tag loss and instrument error. Among the 81 grey seals with adequate data, we found wide variation in their use of space, particularly during the summer period (range of the summer 95% home-range: ~ 5000 km^2^). Although we feel confident that our measures of space-use by female grey seals is representative of the population (*n* = 60), we remain cautious with our estimates of home-range size for males (*n* = 21).

Male grey seals had a larger home-range and core area than females in both the summer and fall seasons. This finding agrees with earlier studies showing that male grey seals where more likely to be directed movers, i.e. exhibiting strong linearity in their movement with long path lengths and exploiting large patches of habitat, while females tended to be residents exploiting smaller patches of habitat closer to Sable Island [35, 63]. Sex-specific differences in movement have been found in other sexually dimorphic mammals: adult male ungulates spend more time walking while females (both lactating and non-lactating) spend more time foraging, and these differences are more pronounced with increasing sexual dimorphism [69]. Strong seasonal sexual segregation has also been demonstrated in the Sable Island grey seal population [34] which likely further contributes toward differences in home-range size. It has been suggested that males may be avoiding areas where females forage to reduce intraspecific competition [51], but this remains to be tested.

Grey seals are sexually size dimorphic with males ~ 1.4 times heavier than females [38]. Generally, given the relationship between body mass and metabolic rate [70], one expects larger individuals to have greater nutritional requirements and thus larger home-ranges [71]. Males also have a larger gut capacity and can consume a greater amount and diversity of low energy dense prey. In contrast females consume fewer prey species of high calorific value [42]. This apparent trade-off for forage quantity over quality may contribute to a larger home-range size in males compared to that of females [72].

We found a negative relationship between size of the core area and body mass with larger individuals of both sexes having a smaller area. Home-range size has been shown to vary inversely with the density of food items available [73], thus individuals exploiting higher quality habitat (i.e., high density of prey) may be expected to have a smaller home-range size. Given that intraspecific competition is likely at foraging patches [36], access to higher quality habitat might be limited to those individuals with greater foraging experience and competitive ability, i.e., older, larger individuals. Smaller and less competitive individuals may therefore be limited to lower quality habitat and potentially experience a greater loss of resources to neighbours [71, 74] leading to the use of larger areas.

Both the home range and core area of males and females contracted in the fall (October to early December) with the approach of the breeding season. Sex-specific changes in the fall distribution [34], movement [35], diving behaviour [39, 40], energy storage [38] and diet [42] of grey seals have been previously reported in this population. Grey seals are capital breeders and body mass is an important determinant of reproductive success in both males and females [75–77]. Thus, seasonal changes in the use of space may indicate that both sexes become more selective in their diets by perhaps targeting higher-quality prey patches to accumulate sufficient energy reserves to support the high energetic demands of reproduction. Beck et al. [42] have shown that the energy density of prey in male grey seals increases from the spring to fall/early winter.

Other studies have examined site and habitat use patterns in pinnipeds, but only at a coarse scale of resolution [5, 14, 32, 78–83]. A primary reason for choosing T-LoCoH to evaluate foraging behaviour and habitat use in this study was its ability to generate temporal indices of site use and allow us to examine movement at a fine spatio-temporal scale. Using the frequency of visits to a hull (a proxy for site fidelity) and the number of locations within a hull (a measure of duration), and along with behavioural and environmental attributes, we have been able to partition the home-range of grey seals according to the nature of its use [54]. Although sex accounted for significant variation in home-range size, it was not a useful predictor of fine-scale site use, suggesting that individual variability may dominate movement behaviour at this fine-scale. Börger et al. [18] found that sex and age in roe deer (*Capreolus capreolus*) became less important predictors of behaviour with a finer scale analysis of home-range size.

Two key findings were apparent in our fine-scale spatio-temporal data. Locations that were grouped into categories 1 and 2 had few return visits, exhibited linear movement behaviour (high eccentricity and low pARS) and tended to occur between Sable Island and offshore banks (e.g., Middle, French, Canso and Sable Bank). Earlier studies have proposed that these areas are important for foraging in grey seals [34–36, 51, 63]. Given their strong directionality, these movements may reflect memory of the direction to foraging areas and/or the use of perceptual cues [84–86]. Although the habitat along these pathways appears to be of little value to grey seals due to the low number of repeat visits, it does provide access to valuable resources and may do so in a manner that minimises energy expenditure or risks of predation [87].

The second key finding concerned locations that were grouped into categories 5 and 6. These locations had many return visits, exhibited non-oriented movement [86] and tended to occur on or close to offshore banks. Non-oriented movement is likely to occur when the behaviour of an animal is influenced by sensory stimuli within its immediate environment, e.g. the presence of prey, leading to frequent turns and slow velocity, i.e. area-restricted search [88–90]. Benthic habitats tend to exhibit lower variability in productivity compared with pelagic environments [4, 91], thus one may expect capital breeders that need to ensure optimal mass gain prior to the breeding season, to exhibit strong site fidelity to specific foraging areas [5, 14, 79, 81–83, 92]. Further, maintaining fidelity to specific areas despite variation in prey availability among years, may confer higher fitness in the long term [5, 11, 12, 14]. Grey seals also spent relatively more time in these high resource habitats during the fall compared to the summer. Thus, together with increasing their foraging effort during the few months prior to the breeding season [38, 40], grey seals also focus their foraging effort in areas that offer high resource value.

One may expect individuals that spend a large proportion of their time at sea in high resource areas to exhibit a high rate of mass gain. Studies on seabirds and pinnipeds have linked positive gains in body mass or lipid content with the location of foraging and the time spent foraging [66, 93]. However, in this study the size of the core area nor the proportion of time spent in locations with a high frequency of return visits were important for predicting body mass gain (a proxy for foraging success). This suggests that for grey seals time spent in apparent foraging behaviour is less important than the quality of the foraging patch or that our measure of apparent foraging overestimates foraging effort. Preliminary data from animal-borne cameras from another study has shown that grey seals from Sable Island rest on the sea-floor during trips to sea (unpublished data, Damian C. Lidgard). Thus, it is possible that locations with a high frequency of return visits were also used for resting. Although records of diving behaviour were available for this study, without data from accelerometers and/or animal-borne cameras, dive records alone are inadequate for distinguishing between resting on the sea floor and foraging.

Understanding how animals use space on a fine-scale provides opportunities to explore individual variation in foraging tactics and the consequences of foraging success on fitness [12, 94, 95]. Further, identifying the drivers of intraspecific variation in movement patterns could improve our ability to predict changes in foraging distribution in response to both natural and man-made environmental forcing [27, 96, 97].

## Conclusion

We have provided a greater understanding of how grey seals use space through incorporating time into home-range analyses. We differentiated areas within the home-range according to the intensity of use and identified high-resource areas. Season, sex and body mass were important predictors of the size of the 95% and core home-range area, however they were less influential in predicting the time spent in areas with high visitation suggesting substantial individual variation in behaviour at this fine-scale. The size of the core-area nor the time spent in areas with high visitation were good predictors of the gain in body mass suggesting behaviours other than foraging were likely occurring in these heavily-used areas.

Thus, although the metrics used in this study were helpful to identify foraging behaviour in grey seals, one must be cautious when making inferences about behaviour from movement data alone [98]. Studies on free-ranging pinnipeds using data collected by head-mounted accelerometers or in situ video will be needed to validate inferences about the at-sea behaviours of these upper-trophic level marine predators.

## Supplementary information

**Additional file 1.** Metadata of grey seals used in the study, 2009 to 2011, and 2013 to 2015.

**Additional file 2.** Parameter estimates of chosen linear mixed-effects model with fixed effects.

**Additional file 3.** Parameter estimates of chosen linear mixed-effects model with fixed effects.

**Additional file 4.** Parameter estimates of chosen linear mixed-effects model with fixed effects.

**Additional file 5.** Descriptive measures of hull visitation and duration of stay within a hull for behavioural categories 1 to 6.

**Additional file 6.** A Parameter estimates of chosen linear mixed-effects model with fixed effects. B Parameter estimates of chosen linear mixed-effects model with fixed effects.

## Data Availability

Data from this study supporting the conclusions of this article are archived with the Ocean Tracking Network (http://oceantrackingnetwork.org/). Access to these data is available from the corresponding author on reasonable request.

## Abbreviations

ER: Evidence Ratio
GPS: Global Positioning System
HMM: Hidden Markov Model
pARS: Probability of Area Restricted Search
T-LoCoH: Time-Local Convex Hull
