# Supplementary material for: Sex-differences in fine-scale home-range use in an upper-trophic level marine predator
Source: Mov Ecol. 2020 Feb 13;8:11. doi: 10.1186/s40462-020-0196-y (PMC7020581; doi:10.1186/s40462-020-0196-y)
Supplement: Supplementary file 1 — Additional file 1. Metadata of grey seals used in the study, 2009 to 2011, and 2013 to 2015. [file 40462_2020_196_MOESM1_ESM.docx]

Table 1 Sex, deployment year and body mass, and percentage initial mass lost per day for grey seals (n = 81), Sable Island, Nova Scotia 2009 to 2011, 2013 to 2015

| Seal ID | Sex | Deployment | | % Initial mass  gained / day |
| --- | --- | --- | --- | --- |
|  |  | Year | Mass, kg |  |
| 2X7 | male | 2009 | 182.0 | 0.30 |
| M120 | male | 2009 | 255.0 | 0.13 |
| M161 | male | 2009 | 257.5 | 0.43 |
| M175 | male | 2009 | 256.0 | 0.06 |
| M691 | male | 2009 | 226.5 | 0.37 |
| 5N3 | male | 2010 | 142.0 | 0.90 |
| 61T | male | 2010 | 201.5 | 0.54 |
| 94J | male | 2010 | 199.0 | 0.60 |
| E113 | male | 2010 | 312.5 | 0.43 |
| M495 | male | 2010 | 196.0 | 0.23 |
| M695 | male | 2010 | 215.5 | 0.21 |
| N001 | male | 2013 | 205.6 | 0.12 |
| N002 | male | 2013 | 202.0 | 0.34 |
| N003 | male | 2013 | 164.8 | 0.38 |
| N007 | male | 2013 | 188.5 | 0.26 |
| 29D | male | 2014 | 206.2 | 0.26 |
| 40B | male | 2014 | 205.0 | 0.22 |
| 4V8 | male | 2014 | 196.4 | 0.29 |
| 79X | male | 2014 | 195.0 | 0.40 |
| 7N2 | male | 2014 | 190.8 | 0.23 |
| X98 | male | 2015 | 188.4 | 0.40 |
| F263 | female | 2009 | 194.5 | - |
| F296 | female | 2009 | 237.0 | - |
| F562 | female | 2009 | 202.5 | - |
| F699 | female | 2009 | 184.0 | - |
| F840 | female | 2009 | 163.5 |  |
| F931 | female | 2009 | 208.5 | - |
| K103 | female | 2009 | 189.0 | - |
| K315 | female | 2009 | 176.0 | - |
| E223 | female | 2010 | 154.0 | - |
| E365 | female | 2010 | 159.0 | 0.18 |
| F 86 | female | 2010 | 184.0 | - |
| F265 | female | 2010 | 140.0 | 0.18 |
| F347 | female | 2010 | 151.5 | - |
| F406 | female | 2010 | 142.0 | 0.33 |
| F446 | female | 2010 | 139.5 | - |
| F465 | female | 2010 | 200.0 | 0.23 |
| F853 | female | 2010 | 219.5 | - |
| F863 | female | 2010 | 149.0 | 0.33 |
| F928 | female | 2010 | 184.0 | - |
| F964 | female | 2010 | 151.5 | 0.23 |
| K436 | female | 2010 | 161.0 | - |
| K475 | female | 2010 | 148.5 | 0.50 |
| E 87 | female | 2011 | 180.0 | 0.06 |
| F104 | female | 2011 | 198.0 | 0.11 |
| F357 | female | 2011 | 172.0 | 0.20 |
| F532 | female | 2011 | 144.0 | 0.22 |
| F719 | female | 2011 | 158.0 | 0.23 |
| K 11 | female | 2011 | 190.0 | 0.12 |
| K 88 | female | 2011 | 186.0 | - |
| S0749 | female | 2011 | 149.0 | 0.08 |
| S0751 | female | 2011 | 125.0 | 0.36 |
| S0753 | female | 2011 | 147.0 | 0.21 |
| S0756 | female | 2011 | 122.0 | 0.26 |
| S0757 | female | 2011 | 139.0 | 0.26 |
| S0758 | female | 2011 | 161.0 | 0.04 |
| 8D4 | female | 2013 | 149.5 | - |
| E 42 | female | 2013 | 175.5 | - |
| E358 | female | 2013 | 183.2 | - |
| E425 | female | 2013 | 172.0 | 0.13 |
| F361 | female | 2013 | 162.0 | 0.09 |
| F503 | female | 2013 | 177.4 | - |
| F857 | female | 2013 | 159.0 | 0.18 |
| N009 | female | 2013 | 165.0 | 0.11 |
| 05V | female | 2014 | 137.0 | - |
| E198 | female | 2014 | 163.4 | - |
| E385 | female | 2014 | 153.0 | 0.24 |
| F188 | female | 2014 | 137.6 | 0.26 |
| F203 | female | 2014 | 143.6 | 0.31 |
| F621 | female | 2014 | 166.6 | 0.11 |
| K327 | female | 2014 | 159.0 | 0.18 |
| E370 | female | 2015 | 158.8 | 0.31 |
| F201 | female | 2015 | 154.6 | 0.30 |
| F204 | female | 2015 | 148.6 | 0.37 |
| F306 | female | 2015 | 140.0 | 0.32 |
| F532 | female | 2015 | 123.8 | 0.20 |
| F875 | female | 2015 | 125.7 | 0.22 |
| F928 | female | 2015 | 207.3 | 0.15 |
| K282 | female | 2015 | 170.8 | 0.14 |
| K390 | female | 2015 | 168.8 | 0.26 |
| K448 | female | 2015 | 125.3 | 0.26 |
