# Supplementary material for: Sex-differences in fine-scale home-range use in an upper-trophic level marine predator
Source: Mov Ecol. 2020 Feb 13;8:11. doi: 10.1186/s40462-020-0196-y (PMC7020581; doi:10.1186/s40462-020-0196-y)
Supplement: Supplementary file 2 — Additional file 2. Parameter estimates of chosen linear mixed-effects model with fixed effects. [file 40462_2020_196_MOESM2_ESM.docx]

Additional File 2 Parameter estimates for linear mixed-effects model for the 95% home-range area for grey seals, Sable Island, Nova Scotia, 2009-2011 and 2013-2015.

| Coefficients | Estimate | SE | t-value | Pr (>\|t\|) |
| --- | --- | --- | --- | --- |
| (~) | 9.54 | 0.05 | 200.7 | <0.001 |
| Sn-Sum | -0.22 | 0.03 | -6.85 | <0.001 |
| Sex-Male | -0.23 | 0.05 | -4.67 | <0.001 |

Fixed effects are season (Sn-Sum) and Sex. N_seals_ = 81
