# Supplementary material for: Sex-differences in fine-scale home-range use in an upper-trophic level marine predator
Source: Mov Ecol. 2020 Feb 13;8:11. doi: 10.1186/s40462-020-0196-y (PMC7020581; doi:10.1186/s40462-020-0196-y)
Supplement: Supplementary file 3 — Additional file 3. Parameter estimates of chosen linear mixed-effects model with fixed effects. [file 40462_2020_196_MOESM3_ESM.docx]

Additional File 3 Parameter estimates for linear mixed-effects models for size of the core area for grey seals, Sable Island, Nova Scotia, 2009-2011 and 2013-2015.

| Coefficients | Estimate | SE | t-value | Pr (>\|t\|) |
| --- | --- | --- | --- | --- |
| (~) | 6.25 | 2.22 | 2.82 | 0.01 |
| Body mass | 1.02 | 0.97 | 1.05 | 0.30 |
| Sn-Sum | -0.44 | 0.13 | -3.42 | <0.001 |
| Sex-Male | 5.69 | 2.51 | 2.27 | 0.03 |
| Sn-Sum * Sex-Male | 0.35 | 0.15 | 2.41 | 0.02 |
| Body mass * Sex-Male | -2.70 | 1.11 | -2.44 | 0.02 |

Fixed effects are body mass, season (Sn-Sum) and sex. The core area is defined as the 30% isopleth based on number of visits to a hull. (N_seals_ = 81, N_obs_ = 126)
