# Supplementary material for: Sex-differences in fine-scale home-range use in an upper-trophic level marine predator
Source: Mov Ecol. 2020 Feb 13;8:11. doi: 10.1186/s40462-020-0196-y (PMC7020581; doi:10.1186/s40462-020-0196-y)
Supplement: Supplementary file 4 — Additional file 4. Parameter estimates of chosen linear mixed-effects model with fixed effects. [file 40462_2020_196_MOESM4_ESM.docx]

Additional File 4 Parameter estimates for linear models for relative body mass gain for grey seals, Sable Island, Nova Scotia, 2009-2011 and 2013-2015.

| Coefficients | Estimate | SE | t-value | Pr (>\|t\|) |
| --- | --- | --- | --- | --- |
| (~) | 0.12 | 0.01 | 12.8 | <0.001 |
| Sex-Male | -0.04 | 0.01 | -3.20 | 0.002 |

### (N_seals_ = 59)
