# Supplementary material for: Sex-differences in fine-scale home-range use in an upper-trophic level marine predator
Source: Mov Ecol. 2020 Feb 13;8:11. doi: 10.1186/s40462-020-0196-y (PMC7020581; doi:10.1186/s40462-020-0196-y)
Supplement: Supplementary file 5 — Additional file 5. Descriptive measures of hull visitation and duration of stay within a hull for behavioural categories 1 to 6. [file 40462_2020_196_MOESM5_ESM.docx]

Additional File 5 Seasonal visits to individual hulls by (a) male and (b) female grey seals for each of the six behavioural categories, Sable Island, Nova Scotia, 2009-2011 and 2013-2015.

(a)

|  |  |  |  |  |  |  |
| --- | --- | --- | --- | --- | --- | --- |
| Behaviour category | Season | Number of visits to a hull / km^2^ | | Duration of visit to a hull, min / km^2^ | |  |
|  |  | median | mad | median | mad | *n* |
|  |  |  |  |  |  |  |
| 1 | S | 0.07 | 0.01 | 9.04 | 0.85 | 9 |
|  | F | 0.06 | 0.02 | 9.21 | 1.34 | 18 |
| 2 | S | 0.08 | <0.01 | 28.0 | 2.01 | 9 |
|  | F | 0.08 | 0.01 | 28.8 | 6.48 | 20 |
| 3 | S | 0.43 | 0.08 | 13.3 | 3.40 | 9 |
|  | F | 0.36 | 0.05 | 15.2 | 3.51 | 20 |
| 4 | S | 0.43 | 0.03 | 50.5 | 6.21 | 9 |
|  | F | 0.43 | 0.06 | 56.8 | 15.5 | 20 |
| 5 | S | 1.33 | 0.16 | 24.7 | 7.85 | 9 |
|  | F | 1.32 | 0.26 | 35.9 | 8.88 | 19 |
| 6 | S | 2.12 | 1.41 | 131.5 | 69.4 | 9 |
|  | F | 2.00 | 1.06 | 99.7 | 44.1 | 18 |

|  |  |  |  |  |  |  |
| --- | --- | --- | --- | --- | --- | --- |
| Behaviour category | Season | Number of visits to a hull / km^2^ | | Duration of visit to a hull, min / km^2^ | |  |
|  |  | median | mad | median | mad | *n* |
|  |  |  |  |  |  |  |
| 1 | S | 0.07 | 0.01 | 8.94 | 2.82 | 37 |
|  | F | 0.07 | 0.02 | 9.76 | 1.81 | 59 |
| 2 | S | 0.08 | 0.01 | 28.6 | 6.87 | 36 |
|  | F | 0.08 | 0.01 | 33.4 | 8.27 | 59 |
| 3 | S | 0.32 | 0.09 | 15.5 | 4.90 | 37 |
|  | F | 0.40 | 0.10 | 17.1 | 3.60 | 59 |
| 4 | S | 0.44 | 0.09 | 59.0 | 18.3 | 36 |
|  | F | 0.47 | 0.08 | 51.3 | 14.1 | 57 |
| 5 | S | 1.21 | 0.24 | 38.0 | 11.7 | 32 |
|  | F | 1.33 | 0.27 | 38.4 | 9.23 | 57 |
| 6 | S | 1.49 | 0.43 | 102.1 | 25.5 | 24 |
|  | F | 1.75 | 0.58 | 108.1 | 31.3 | 53 |

(b)

Measures of hull visitation are the normalised number of visits per km^2^ and duration of stay within a hull, min / km^2^. See text for description of behavioural categories. S = summer; F = fall. mad = median absolute deviation. n = number of individual seals that exhibited the behaviour common to the behavioural category
